# Expediting the Rehabilitation of Severely Resorbed Ridges Using a Combination of CAD-CAM and Analog Techniques: A Case Report

**DOI:** 10.3390/medicina60020260

**Published:** 2024-02-02

**Authors:** Carlos A. Jurado, Francisco X. Azpiazu-Flores, Chin-Chuan Fu, Silvia Rojas-Rueda, Gerardo Guzman-Perez, Franciele Floriani

**Affiliations:** 1 Department of Prosthodontics, College of Dentistry and Dental Clinics, The University of Iowa, Iowa City, IA 52242, USA; 2 Department of Restorative Dentistry, Gerald Niznick College of Dentistry, University of Manitoba, Winnipeg, MB R3E 3N4, Canada; 3 Center for Implant, Esthetic, and Innovative Dentistry, Department of Prosthodontics, Indiana University School of Dentistry, Indianapolis, IN 46202, USA; 4 Department of Restorative Dentistry, University of Alabama at Birmingham School of Dentistry, Birmingham, AL 35233, USA; 5 School of Dentistry, Universidad Javeriana, Bogota 110231, Colombia; 6 Department of Periodontology, Centro Educative Multidisciplinario en Rehabilitation Oral (CEMRO), Morelia 58880, Mexico; 7 Department of Periodontology, Quetzalcoatl University, Irapuato 36615, Mexico

**Keywords:** CAD-CAM, dental implants, residual ridge resorption

## Abstract

With the life expectancy increasing, there is a growing need for prosthetic dental treatments to restore the oral health, function, and quality of life of edentulous patients. Presently, only a few articles are available describing the oral rehabilitation of patients with severely resorbed ridges with milled complete dentures. This clinical case report provides a straightforward protocol consisting of a combination of analog and digital techniques for the rehabilitation of edentulous patients with severely resorbed ridges with milled fixed and removable complete dentures. This technique permits the minimization of the number of appointments, improves patient comfort, allows for the digital archiving of important clinical data, and permits the manufacture of prostheses with improved mechanical properties. These favorable outcomes were achieved by using the patient’s existing PMMA complete denture as a custom tray for a final impression with light-bodied Polyvinylsiloxane. Subsequently, the resulting models were digitized, and a digital complete denture was designed and manufactured in an expedited manner using CAD-CAM techniques. Therefore, this case report highlights the potential of CAD/CAM technology to predictably restabilize oral functions and improve patients’ quality of life.

## 1. Introduction

Residual ridge resorption is the chronic, progressive reduction in the quantity and quality of the alveolar bone occurring after teeth are extracted [1]. The progression of this phenomenon is accompanied by unfavorable intraoral and extraoral changes including compromised facial esthetics, forward positioning of the mandible, compromised maxillomandibular relationships, and severe reductions in denture-bearing surfaces [1,2]. Factors such as nutritional deficiencies, anatomical factors, and the use of complete dentures with unsatisfactory occlusal relationships have been identified as exacerbating factors for this phenomena [3]. Until the introduction of dental endosteal implants, the only restorative option available for edentulous patients consisted of removable complete dentures which, despite all the advances related to dental materials and occlusal concepts, were only capable of restituting masticatory function to some extent [4].

Various denture base resin materials have been developed and are employed in dentistry for complete denture fabrication. Polymethyl methacrylate (PMMA), introduced by Dr. Walter Wright in 1937, stands out as the most widely accepted material for this purpose [5]. While PMMA exhibits excellent physical and mechanical properties, it has some limitations, necessitating improvements in its strength. Complete denture fractures often result from impact and flexural forces [6]. Therefore, both flexural and impact strengths are key parameters for comparing the performance of denture base resin materials. The fabrication methods employed significantly influence denture impact strength. Conventional techniques often result in internal defects, such as porosity, and lead to shrinkage, directly affecting the mechanical properties of dentures [7,8]. 

Traditional protocols for rehabilitating edentulous patients with fixed complete dentures consisted of 4–6 endosteal implants placed between the mental foramina [9]. Traditionally, this implant configuration was used to retain a prosthesis comprising a cast-metal substructure which was layered with a heat-cured polymethylmethacrylate (PMMA) denture base and artificial teeth [10,11]. In response to the challenges posed by conventional fabrication protocols, the computer-aided design/computer-aided manufacturing (CAD/CAM) technique was introduced. This technique can involve milling or printing the denture base and artificial teeth of the prosthesis separately or both elements from the same blank of material [12]. With the advent of contemporary data acquisition and imaging technologies, these advanced surgical procedures can be planned completely digitally [13,14], and complete arch prostheses can be fabricated from start to finish using modern computer-aided design and computer-aided manufacturing (CAD-CAM) workflows [15,16].

At present, only a few studies have evaluated the surface characteristics of the CAD-CAM denture base resins available on the market. Current research suggests that the milling technique yields complete denture bases with superior accuracy and complete denture base fitting [17]. Other studies have reported significant differences in roughness and contact angle between milled resins in comparison to conventional PMMA resins, favoring digital techniques [18,19]. In addition, contemporary protocols for milled complete dentures may shorten chairside time by reducing clinical occlusal adjustment [20]. 

For the digital manufacturing of complete dentures, a method for achieving pink and white esthetics involves milling the denture base and denture teeth separately using acrylic resin disks of distinct colors and subsequently bonding them together [21]. This approach has demonstrated reliability, and the bond strength between the milled denture teeth and base is comparable, or even superior, to that observed between heat-polymerized acrylic resin and artificial teeth [22]. CAD-CAM technology permits the expedition of the design and manufacture of complex dental prostheses while minimizing the extent of manual labor involved [23,24]. These technologies have been successfully used to rehabilitate elderly patients and patients with complex craniofacial and systemic conditions needing extensive dental treatment [25] and have permitted the development of alternative diagnostic methods to visualize and validate the occlusal relationships of future prostheses intraorally [26].

This case report presents the successful rehabilitation of a patient with severely resorbed maxillary and mandibular ridges with a combination of removable and fixed complete dentures designed and fabricated using a combination of analog techniques and CAD-CAM technology. The clinical workflow presented is an alternative to traditional planning and surgical procedures that permits the completion treatment in a shorter period since both the treatment and the definitive prosthesis are fabricated using contemporary digital technologies.

## 2. Materials and Methods

**Figure 1:**
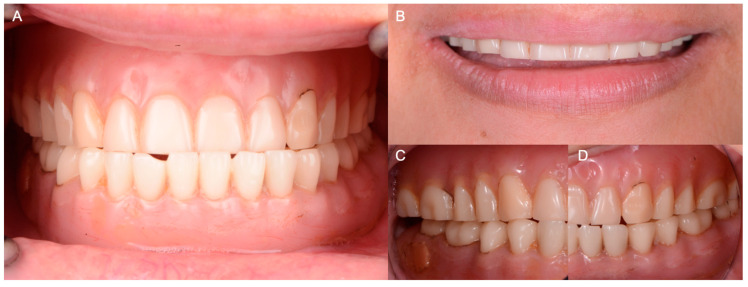
Initial complete dentures. (**A**) Occlusion frontal view; (**B**) smile; (**C**) right side view; (**D**) left side view.

A 45-year-old female patient presented with the chief complaint of needing new complete dentures. At the time of the initial consultation, the patient reported that she had lost all her teeth from a combination of caries and periodontal disease. At that time, the patient had upper and lower complete dentures which were fabricated 15 years ago. She expressed her dislike for the appearance and function provided by the prostheses. When the prostheses and residual ridges were examined intraorally, due to the pattern of ridge resorption and the loss of vertical dimension of the worn prostheses, the maxillomandibular relationships of the patient became prognathic, thus compromising the functionality and esthetics of the prostheses; furthermore, significant discoloration of the denture bases and fracture of the artificial teeth were also noted (Figure 1). 

**Figure 2:**
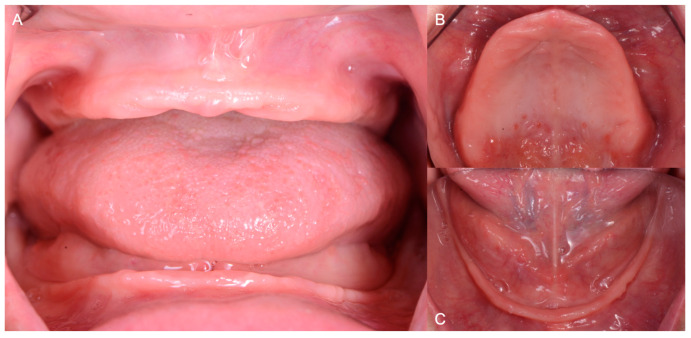
Initial intraoral situation. (**A**) frontal view; (**B**) maxillary occlusal view; (**C**) mandibular occlusal view.

When the patient’s medical history was evaluated, no personal or family history of significant systemic issues or medical conditions was reported. Moreover, the patient did not report taking any medication that could affect the treatment outcome. Subsequently, an intraoral clinical evaluation was performed, and the residual bone height was evaluated. Due to having a residual vertical bone height of 10 mm or less at the least vertical height of the mandible, a loss of anterior labial and posterior buccal vestibules, high frenum insertions, minimal attached tissues and an unfavorable palatal vault morphology, the patient was classified as a severely compromised edentulous patient according to the ACP classification for complete edentulism (Figure 2).

Different treatment options including removable upper and lower complete dentures, maxillary and mandibular implant overdentures, and fixed-implant-supported maxillary and mandibular prostheses were presented and discussed with the patient. Due to financial factors, the patient selected the treatment plan consisting of an upper complete denture and a lower fixed complete denture. The patient was informed that an initial full set of complete dentures was needed to define the vertical dimension, lip support, esthetics, and phonetics that will direct the final prostheses. After discussing the steps involved in the fabrication of the protheses, the patient was offered the option of having the prostheses fabricated using CAD/CAM technology to expedite the progression of the treatment, and she accepted. 

**Figure 3:**
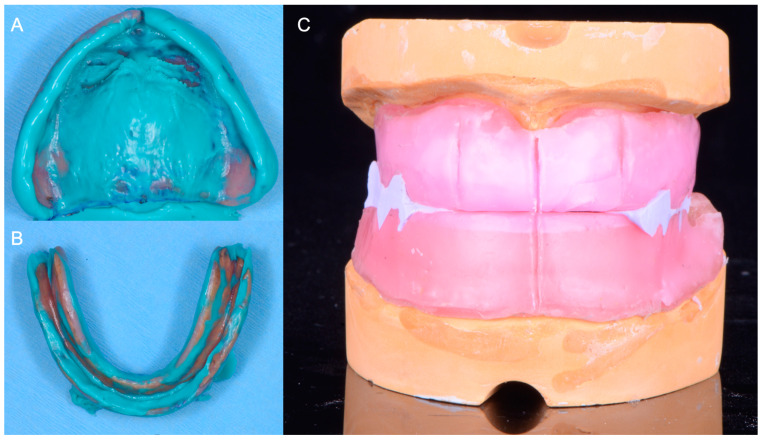
Final impressions and maxillomandibular relationship records. (**A**) Maxillary final impression using patient’s maxillary complete denture as a customized impression tray; (**B**) mandibular final impression; (**C**) master casts related using maxillo-mandibular relationship records.

Final impressions were made with a polyvinyl siloxane (PVS) impression material (Aquasil Impression Material, Dentsply Sirona, Charlotte, NC, USA), using her existing maxillary and mandibular complete dentures as customized impression trays. Subsequently, conventional maxillomandibular relationship records were made with photopolymerized record bases (Triad VLC Denture Base Material, Dentsply Sirona, Charlotte, NC, USA), and occlusion rims were made using pink baseplate wax (Hygenic Wax, Coltene, Cuyahoga Falls, OH, USA). At this stage, the centric relation record was obtained with vinyl siloxane material (Regisil, Dentsply Sirona, Charlotte, NC, USA) (Figure 3).

**Figure 4:**
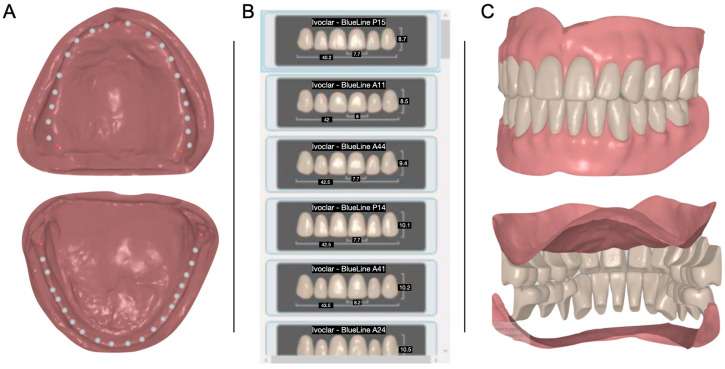
Digital design of the complete dentures. (**A**) Tooth location for maxilla and mandible; (**B**) selecting tooth shape; (**C**) digital tooth arrangement for the dentures.

The resulting master casts were mounted in a semi-adjustable articulator (Panadent, Colton, CA, USA), and their special relationship was digitally scanned. Subsequently, the artificial teeth mold and location were digitally arranged using the occlusion rims as a reference (Avadent, Scottsdale, AZ, USA) (Figure 4).

**Figure 5:**
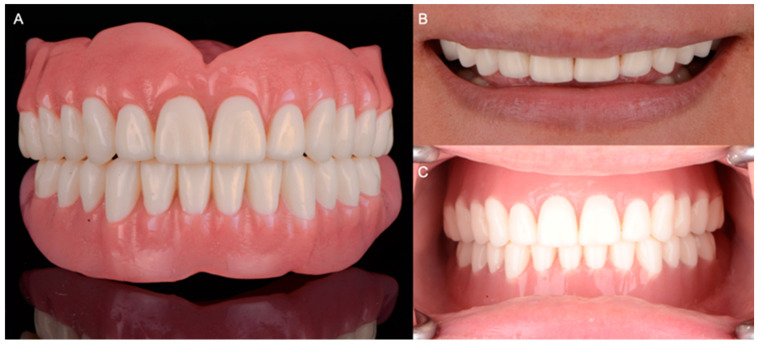
Final milled complete dentures. (**A**) Frontal view of maxillary and mandibular milled complete dentures; (**B**) patient smiling with dentures; (**C**) maxillary and mandibular complete dentures in centric occlusion.

Maxillary and mandibular complete dentures were milled from high-density polymethylmethacrylate resin (PMMA) (Avadent, Scottsdale, AZ, USA) and tried intraorally. Subsequently, the central relation, vertical dimension, lip support, esthetics, phonetics, and smile line were evaluated and adjusted until the patient and clinician were satisfied with the results (Figure 5).

**Figure 6:**
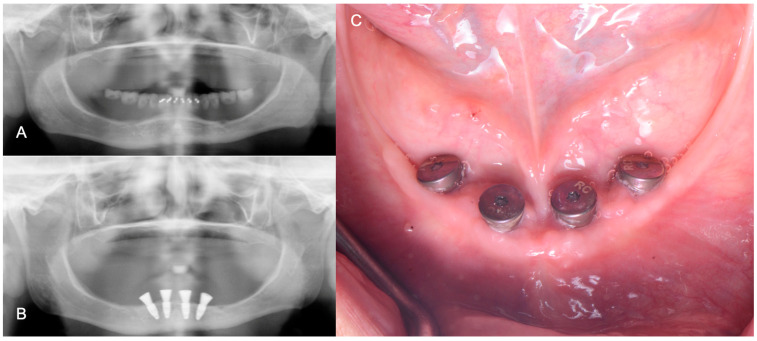
Before and after implant placement radiographs and clinical photograph. (**A**) Initial panoramic radiograph; (**B**) panoramic radiograph after implant placement; (**C**) occlusal view of mandibular implants.

After the previous parameters were validated intraorally, the patient approved and signed an informed consent document prior to the surgery, and four mandibular implants (Straumann 4.1 RC, Basel, Switzerland) were placed between the mental foramina using the contours of the milled mandibular complete denture as a reference (Figure 6).

**Figure 7:**
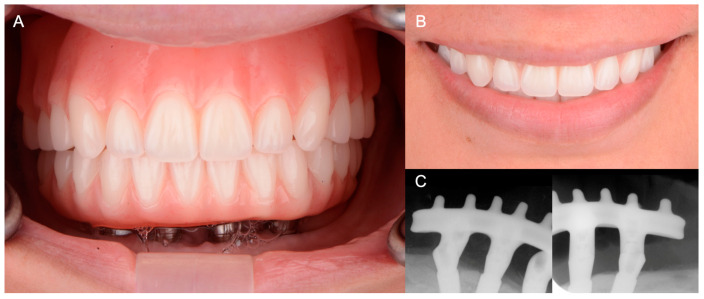
Final prostheses. (**A**) Frontal view of final maxillary complete denture and fixed mandibular complete denture; (**B**) smile with the final maxillary and mandibular prostheses; (**C**) periapical radiographs of the mandibular implants and implant superstructure on the day of delivery.

After 4 months, the mandibular implants were assessed and deemed adequate to proceed with the fabrication of the definitive mandibular prosthesis. The final maxillary complete denture and mandibular fixed complete denture were milled (Avadent, Scottsdale, AZ, USA) following the same digital planning used for the first set of complete dentures. Similarly, esthetics, function, smile line, lip support and phonetics were evaluated. At this stage, both the patient and clinicians were satisfied with the results (Figure 7).

After delivery, the patient was instructed to return to the dental office after 24 h, 3 days, and 1 week to monitor the evolution of the dental treatment. Complete denture fitting was evaluated and adjusted if needed, and the patient’s oral hygiene was reassessed for the mandibular prosthesis. Furthermore, occlusion and the patient’s overall satisfaction were also evaluated, both with favorable results. After that, the patient was requested to come every 6 months for prostheses and oral hygiene evaluations.

## 3. Results

**Figure 8:**
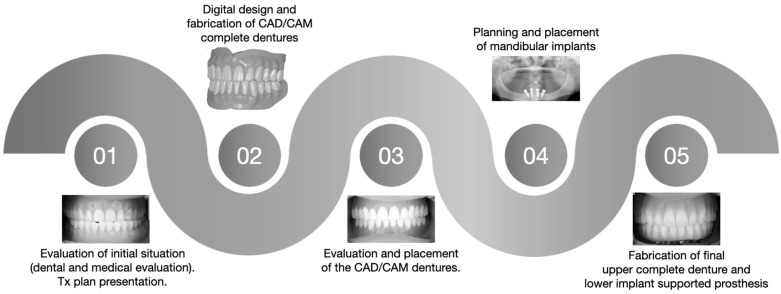
Flowchart describing the clinical workflow implemented for this treatment.

A combination of analog and CAD-CAM techniques were used to attain precision in the final milled maxillary and mandibular prostheses in an uncomplicated fashion and with a reduced number of appointments. Initially, the first analog impressions were obtained using the existing complete dentures as customized impression trays. Subsequently, the digital planning of the implant positions was defined using the contours of the first set of milled prostheses as a reference. A flowchart of the workflow performed for this treatment can be seen in Figure 8.

## 4. Discussion

The presented technique presents several advantages compared to traditional protocols for complete denture therapy. These advantages include a reduction in the number of appointments, improved patient comfort, the ability to carry out digital archiving in case remakes are required, and improved treatment predictability by manufacturing prostheses with highly dense materials. Furthermore, the technique presented permits clinicians to circumvent errors related to the intraoral scanning of edentulous ridges. Contemporary research suggests that scanning a completely edentulous arch remains challenging due to limitations such as the presence of mobile mucosa [27], issues with light reflection [28], the inter-implant distance, and the scanning protocol [29]. Additionally, the lack of anatomic landmarks, such as teeth, poses a challenge for the registration and superimposition of images recorded by the intraoral scanner [30]. A previous study evaluated the flexural strength of three different complete denture materials (heat-cured PMMA, milled PMMA, and 3D-printed denture base photopolymer). As a result, a flexural strength of 80.79 (±7.64 MPa) for heat-cured analog resins was found, and a flexural strength of 110.23 (±5.03 MPa) for the CAD-CAM-milled PMMA block (Ruthinium Disc, Dental Manufacturing Spa) was identified. The 3D-printed Nextdent denture base specimens showed very similar flexural strength (87.34 ± 6.39 MPa), even though they were printed using an SLA printer and polymerized for 20 min with a light box (Moonlight, VertySystem, Vicenza, Italy) [31]. When compared with a conventional workflow, the digital complete dentures required a statistically shorter clinical time, with 205 to 233 min saved. The outcomes for patient satisfaction and oral-health-related quality of life were similar between conventional, milled, and 3D-printed complete dentures [32].

Research suggests that digital methods for designing and manufacturing milled dentures result in dental prostheses with improved flexural strength, flexural modulus, yield strength, toughness, surface properties, and color stability when compared to 3D-printed complete dentures [33]. Clinically, both the milled and 3D-printed complete dentures performed better than conventional dentures in terms of retention [34], but the current literature does not support the use of 3D-printed dentures for the long term or for patients with high esthetic demands. The approach outlined in this article represents an evolution of the concept toward a hybrid analog–digital workflow. With this approach, all information can be consistently maintained digitally, and a final implant-supported restoration, mirroring the occlusion tested with the provisional diagnostic dentures, can be delivered to the patient. Additionally, this protocol streamlines the clinical process and gives flexibility to the clinician to produce either a CAD/CAM or a 3D-printed hybrid prosthesis depending on the situation. Furthermore, the increasing prevalence of milling machines in dental labs and offices holds promising potential for rapidly producing prostheses at reasonable cost, increasing patient satisfaction and accuracy, and reducing chairside time by reducing clinical occlusal adjustment with highly predictable denture base fitting. This innovative approach to prosthetic dentistry showcases the potential for advanced digital techniques to bring about precision and positive outcomes in patient care. Further research and clinical studies are warranted to explore the broader applicability and long-term effectiveness of milled complete dentures in diverse patient populations.

A take-home message after concluding this treatment is to carefully evaluate the patient’s initial clinical situation, esthetic expectations, and functional demands prior to starting any extensive prosthetic treatment. Moreover, the inclusion of novel technology may offer some benefits such as expediting the time process and provide more predictable outcomes since occlusion can be clinically validated intraorally before the definitive prostheses are made, and in the case that non-ideal outcomes are encountered, they can be easily redesigned and remade. In addition, this clinical report presents limitations related to the equipment required to execute the treatment, which is not available in all dental clinics or dental laboratories. Furthermore, the authors acknowledge that the present clinical report represents an alternative treatment modality for a specific clinical scenario which may not be applicable for other clinical situations, such as those involving terminal dentitions needing treatment or patients with resorbed ridges requiring extensive regenerative therapy. However, despite these limitations, the present clinical report can be used as a reference in similar clinical situations or as a reference for the development of alternative treatment protocols by adapting the CAD-CAM or analog procedures involved in the protocol.

## 5. Conclusions

Within the limitations of this clinical case report, it can be concluded that the use of milled complete dentures as a diagnostic and functional appliance permitted the predictable rehabilitation of the patient while reducing the overall clinical time. Furthermore, the presented technique implemented the patient’s existing prostheses as an impression device to record the denture-bearing tissues, thus taking advantage of this appliance. Finally, the presented technique is an alternative approach that can be adapted to other situations by means of modifying the CAD-CAM or analog procedures involved. 

## Data Availability

The data presented in this study are available on request from the corresponding author.
